# A combination of supraglottic airway and bronchial blocker for one-lung ventilation in infants undergoing thoracoscopic surgery

**DOI:** 10.1016/j.heliyon.2023.e13576

**Published:** 2023-02-09

**Authors:** Junlin Lv, Xiaoying Ding, Jing Zhao, Huijuan Zhang, Jiaojiao He, Lei Ma, Jianrui Lv

**Affiliations:** a Department of Anesthesiology, Second Affiliated Hospital of Xi'an Jiaotong University, Xi'an, Shaanxi, China; b Department of Orthodontics, College of Stomatology, Xi'an Jiaotong University, Xi'an, Shaanxi, China

**Keywords:** Supraglottic airway, Bronchial blocker, One-lung ventilation, Anesthesia, Infant

## Abstract

**Objectives:**

One-lung ventilation (OLV) for children under the age of two years is difficult. The authors hypothesize that a combination of a supraglottic airway (SGA) device and intraluminal placement of a bronchial blocker (BB) may provide an appropriate choice.

**Design:**

A prospective method-comparison study.

**Setting:**

Second Affiliated Hospital of Xi'an Jiaotong University, China.

**Participants:**

120 patients under the age of two years undergoing thoracoscopic surgery with OLV.

**Interventions:**

Participants were randomly assigned to receive intraluminal placement of BB with SGA (n = 60) or extraluminal placement of BB with endotracheal tube (ETT) (n = 60) for OLV.

**Measurements and main results:**

The primary outcome was the length of postoperative hospitalization stay. The secondary outcomes were the basic parameters of OLV and investigator-defined severe adverse events. The postoperative hospitalization stay was 6 days (interquartile range, IQR 4–9) in SGA plus BB group compared with 9 days (IQR 6–13) in ETT plus BB group (*P* = 0.034). The placement and positioning duration of SGA plus BB was 64 s (IQR 51–75) compared with 132 s (IQR 117–152) of ETT plus BB (*P* = 0.001). The values of leukocyte (WBC) and C-reactive protein (CRP) of SGA plus BB group on the first day of post-operation were 9.8 × 10^9^/L (IQR 7.4–14.5) and 15.1 mg/L (IQR 12.5–17.3) compared with 13.6 × 10^9^/L (IQR 10.8–17.1) and 19.6 mg/L (IQR 15.0–23.5) of ETT plus BB group (*P* = 0.022 and *P* = 0.014).

**Conclusion:**

There were few if any significant adverse events in the intervention group (SGA plus BB) for OLV in children under the age of two years, and this method seems worthy of clinical application. Meanwhile, the mechanism for this novel technique to shorten the length of postoperative hospitalization stay needs to be further explored.

## 1 Introduction

Implementing one-lung ventilation (OLV) for children under the age of two years is difficult. These lective endobronchial intubation with a tracheal tube does not allow easy alternations between one and two lung ventilation [1,2]. A double lumen tube (DLT) can be used for OLV in children older than eight years. In younger children, a bronchial blocker (BB) is commonly used and is placed either inside (>two years) or outside (<two years) the endotracheal tube (ETT) [3,4]. However, the ETT with a BB may not be correctly placed, which prolongs the effective anesthesia and operation time [5,6]. Complications associated with the ETT and BB malposition result in an urgent problem to be solved to ensure sufficient ventilation, reduce postoperative airway complications, and promote rapid recovery for small children involved in OLV.

The novel combination of the supraglottic airway (SGA) and BB has been implemented in adult thoracic surgery requiring OLV, which ensures effective ventilation and results in few postoperative airway complications [[7], [8], [9]]. However, whether the combination of SGA and BB can effectively solve the OLV problem for children under the age of two years has not been investigated.

In our clinical trials, we aimed to establish whether the combined use of the SGA and BB would be superior to extraluminal placement of BB for OLV in children under the age of two years.

## 2 Methods

### 2.1 Study design and participants

A randomized controlled clinical trial was performed. The trial protocol was approved by the medical ethics committee of the Second Affiliated Hospital of Xi'an Jiaotong University in China (Ethical Approval Number 20190030) and the *Chinese Clinical Trial Registry* (ChiCTR2000030213). Written informed consent was obtained from all legal representatives of the patients enrolled in the beginning of the study. The study was performed in accordance to the Declaration of Helsinki and Good Clinical Practice.

A total of 120 patients who were classified as I–II on the health rating scale according to the standards and guidelines of the American Society of Anaesthesiologists (ASA) and underwent thoracoscopic surgery under OLV for an estimated operative time of about 2 h from March 2020 to July 2021 were enrolled in this prospective study. The patients were randomly divided into two parallel groups: intraluminal placement of BB with SGA group, and extraluminal placement of BB with ETT group. Inclusion criteria were patients under two years old or children with a body mass index (BMI) of 15–30 kg/m^2^ undergoing thoracoscopic surgery for congenital pulmonary airway malformation (CPAM) and required OLV. The exclusion criteria were as follows: known compression of the trachea or the left/right main bronchus; anticipated intubation difficulties (Mallampati score ≥3); conversion to thoracotomy; and an operative time over 3 h.

### 2.2 Randomization and masking

The study coordinator performed block randomization (10 in each block). The randomization list was computer generated. Patients were randomly assigned into groups (1–3 h before surgery, usually on the morning of the procedure) before receiving CPAM surgery. Surgeons and assistants who performed in CPAM surgery procedures were masked to group assignment. Investigators who collected demographic or procedure-related data or participated in the assessment of post-operative complications were also masked to group assignment. The statistician who analyzed the data was unaware of the group assignments. Patients were not masked to trial-group assignment.

### 2.3 Procedures

Anesthetic monitoring, including electrocardiography, pulse oximetry, capnography, and non-invasive blood pressure monitoring, was conducted prior to anesthesia induction and during the surgery. After the induction of anesthesia, an arterial line was established for determining the baseline of arterial blood gas, blood pressure, and heart rate. All patients were anesthetized by a chief resident who is experienced in the use of the SGA and BB.

For the induction of anesthesia, propofol (2.5 mg/kg), fentanyl (3 μg/kg), and *cis*-atracurium (0.2 mg/kg) were injected intravenously. General anesthesia was maintained with target controlled infusion using a Graseby™ 3500 syringe pump (Smith Medical MD, Inc., USA) of propofol at 8.0 μg/ml (plasma concentration) and with continuous infusion of 2.0 μg/kg/h fentanyl and 0.1 mg/kg/h *cis*-atracurium.

**Fig. 1:**
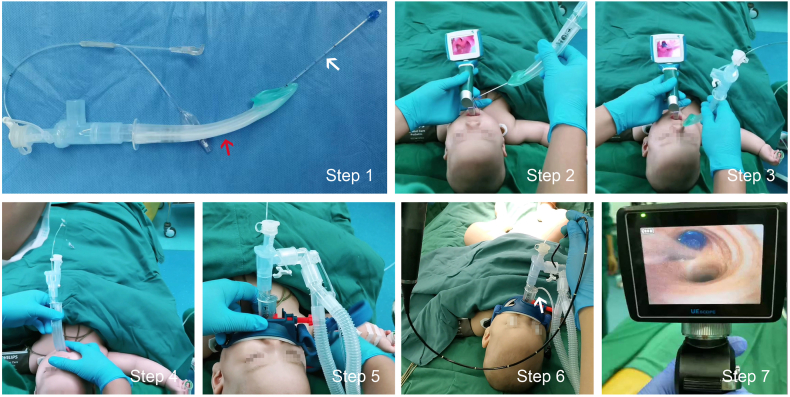
Illustration of the specific operation procedure of the combination of supraglottic airway and intraluminal placement of bronchial blocker. Step 1: The BB (shown by the white arrow) well lubricated with paraffin oil traveled through the SGA ventilation tube (indicated by the red arrow) to form a combination; Step 2: The glottis was exposed through the visual laryngoscope and the previously formed combination was inserted into the trachea as a whole as in a regular endotracheal intubation operation; Step 3: The anterior cuff of the BB passed through the glottis with the SGA remaining outside the mouth; Step 4: The SGA was placed in the appropriate position of the pharyngeal cavity to ensure ventilation, leading the BB to theapproximate location of the tracheal carina; Step 5: A fixator was used to guarantee the position of the SGA; Step 6: A gastric tube of 8G highlighted by the white arrow was placed through the esophageal drainage tube of the SGA to lower intragastric pressure and thus reduce occurrence of reflux aspiration; Step 7: The 2.8-mm fiberoptic bronchoscope was inserted through the SGA and the depth of the BB was adjusted until the location was satisfactory reached under direct vision. Abbreviations: SGA, supraglottic airway; BB, bronchial blocker. (For interpretation of the references to colour in this figure legend, the reader is referred to the Web version of this article.)

In the SGA plus BB group, the BB (EBT0105, TAPPA, China) was well lubricated with paraffin oil throughout the ventilation tube of the SGA (i-gel, Intersurgical, UK) to form a combination of the two devices which was then inserted into the glottis as a whole under direct visual control of a visual laryngoscope (VL300 M, UE, China) followed by the SGA inserted into the oropharynx to allow controlled ventilation with no leakage, the tidal volume fluctuating at 10% of the set value, and the airway pressure below 30 cmH_2_O. After that, a gastric tube of 8G was placed through the esophageal drainage tube of the SGA to lower intragastric pressure and thus reduces the occurrence of reflux aspiration. Finally, the 2.8-mm fiberoptic bronchoscope (TIC-SD-I, UE, China) was inserted through the SGA to position the BB in the bronchus to be blocked (Fig. 1).

In the ETT plus BB group, the ETT (I-A; WORK, China) and extraluminal placement of BB (EBT0105, Tappa, China) were inserted into the glottis through the visual laryngoscope (VL300M, UE, China), and then the 2.8-mm fiberoptic bronchoscope (TIC-SD-I, UE, China) was inserted through the ETT to position the BB in the bronchus to be blocked.

The patient was ventilated with a pressure-controlled volume guaranteed mode to achieve a tidal volume of 10 ml/kg (Primus, Dräger, Germany), aninspiratory/expiratory (I/E) ratio of 1:1.5, a respiratory rate of 30–35 breaths/min, and positive end-expiratory pressure (PEEP) of 3 cmH_2_O.The respiratory rate was adjusted to keep an end-tidal CO_2_ below 80 mmHg which was predefined as hypercapnia in this clinical scenario. If the partial pressure of CO_2_ was higher than 80 mmHg, the operation would be suspended to implement double lung ventilation until the end-tidal CO_2_ below 80 mmHg. After patients were positioned to the lateral decubitus, the cuff position of the BB was checked again. Evaluation of lung collapse was performed by a thoracic surgeon blinded to the group assignment. Collapse of the lung was assessed according to the lung collapse score: 10 = complete collapse, 0 = no collapse [10].

### 2.4 Outcome measurements

The primary outcome measurement was the length of postoperative hospitalization stay. The secondary outcomes were the placement and positioning duration of SGA or ETT plus BB; the mean arterial pressure (MAP) and heart rate (HR) immediately after insertion of SGA plus BB or ETT plus BB; the peak airway pressure and tidal volume 5 min after OLV; the quality of lung isolation (evaluated by the surgeon blinded to the group assignment according to the lung collapse score); the duration from the end of surgery to the removal of laryngeal mask or endotracheal tube; the leukocyte count (WBC) and the value of C-reactive protein (CRP) on the first day of post-operation. Recorded adverse events included the incidence of hypoxemia or hypercapnia during surgery, incidences of laryngospasm, stridor and airway obstruction during awakening, and the incidences of hoarseness within three days after operation.

### 2.5 Statistical analysis

A relative between-group difference of 40% in the primary outcome measure was considered to be clinically relevant for changing clinical practice. A sample size of 112 neonates was required for a 90% chance of detecting an absolute difference of 10% points in the primary outcome at a two-sided significance level of 5%.

The quantitative variables were presented as the median (interquartile range, IQR) and the Mann-Whitney *U* test was used to compare results. Qualitative variables were presented as frequency (percentage) and compared between the two groups with use of the Chi-square or Fisher's exact test. Effect sizes were reported as relative risk (RR) with a 95% confidence interval (CI). All tests were two-sided, and a *P* value < 0.05 was considered statistically significant. Data were analyzed with the Stata (version 12.0) statistical software.

## 3 Results

### 3.1 Characteristics of the patients

**Fig. 2:**
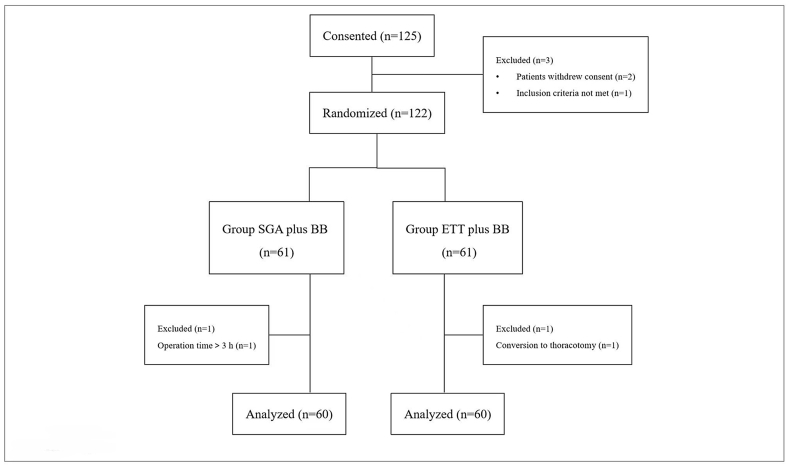
Consolidated standards of reporting trials flow diagram. Abbreviations: SGA, supraglottic airway; BB, bronchial blocker; ETT, endotracheal tube.

Among the 125 pediatric patients who underwent thoracoscopic surgery under OLV, three did not meet the inclusion criteria or refused to participate. Therefore, 122 patients were included in this study, with 61 patients in each group, BB with SGA or BB with ETT. Due to prolonged operation time or changes in surgical methods, 60 cases in each group were finally analyzed (Fig. 2). The general conditions of the patients before surgery were shown in Table 1. There was no significant difference in age, sex, BMI, ipsilateral lung conditions, surgery duration, OLV duration, and blood loss in surgery between the two groups.

**Table 1:** Baseline characteristics of patients.

	SGA plus BB n = 60	ETT plus BB n = 60
Age (months)	6 (2–9)	6 (3–11)
Sex		
Boys	36 (51.4%)	35 (52.2%)
Girls	34 (48.6%)	32 (47.8%)
BMI (kg/m^2^)	21.2 (18.3–25.1)	20.8 (18.4–23.5)
Ipsilateral lung		
Left	33 (47.1%)	34 (50.7%)
Right	37 (52.9%)	33 (49.3%)
ASA		
1	67 (95.7%)	65 (97%)
2	3 (4.3%)	2 (3%)
Surgery duration (min)	99 (81–114)	102 (85–123)
OLV duration (min)	87 (75–100)	90 (79–102)
Blood loss in surgery (mL)	16 (14–19)	17 (15–19)

Values are expressed as median (25%–75% interquartile range) or number of patients (n, %).

Abbreviations: SGA, supraglottic airway; ETT, endotracheal tube; BB, bronchial blocker; BMI, body mass index; ASA, American Society of Anesthesiologists; OLV, one lung ventilation.

### 3.2 Primary outcome

The length of postoperative hospitalization stay was 6 days (IQR 4–9) in the SGA plus BB group compared with 9 days (IQR 6–13) in ETT plus BB group (Table 2, *P* = 0.034).

**Table 2:** The primary and secondary outcomes.

	SGA plus BB n = 60	ETT plus BB n = 60	Median difference (95% CI)	*P* Value*
Primary outcome				
Postoperative hospitalization stay (days)	6 (4–9)	9 (6–13)	3 (2–6)	0.034
Secondary outcomes				
Placement and positioning duration of SGA or ETT plus BB (s)	64 (51–75)	132 (117–152)	69 (51–85)	0.001
Mean arterial pressure (mmHg)^a^	51 (47–56)	52 (49–55)	0 (–3–2.5)	0.361
Heart rate (bmp)^a^	129 (118–141)	130 (119–143)	0 (−2.5–3)	0.292
Peak airway pressure (cmH_2_O)^b^	19 (15–22)	20 (16–26)	0 (−1.5–2)	0.411
Tidal volume (mL)^b^	69 (54–83)	67 (51–82)	1 (–2–2)	0.384
Lung isolation score	9 (8–10)	9 (7–10)	0 (–1–1.5)	0.625
Duration from the end of surgery to the removal of laryngeal mask or endotracheal tube (min)	9 (7–12)	11 (8–14)	2 (0–3)	0.167
Value of WBC (10^9^/L)	9.8 (7.4–14.5)	13.6 (10.8–17.1)	4.0 (2.7–6.2)	0.022
Value of CRP (mg/L)	15.1 (12.5–17.3)	19.6 (15.0–23.5)	4.3 (2.5–6.7)	0.014

Values are expressed as median (25%–75% interquartile range).

*P values indicate differences between SGA plus BB and ETT plus BB patients. *P* < 0.05 was considered statistically significant.

Abbreviations: SGA, supraglottic airway; ETT, endotracheal tube; BB, bronchial blocker; WBC, leukocyte; CRP, C-reactive protein.

a The mean arterial pressure (MAP) and heart rate (HR) were recorded immediately after insertion of SGA plus BB or ETT plus BB.

b The peak airway pressure and tidal volume (VT) were recorded at 5 min after one lung ventilation.

### 3.3 Secondary outcomes and safety

The placement and positioning duration of SGA plus BB was 64 s (IQR 51–75) compared with that of ETT plus BB which was 132 s (IQR 117–152, *P* = 0.001, Table 2). The WBC value of the SGA plus BB group on the first day of post-operation was 9.8 × 10^9^/L (IQR 7.4–14.5) compared with 13.6 × 10^9^/L (IQR 10.8–17.1) of the ETT plus BB group (*P* = 0.022, Table 2). The amount of CRP in the SGA plus BB group on the first day of post-operation was 15.1 mg/L (IQR 12.5–17.3) compared with 19.6 mg/L (IQR 15.0–23.5) in the ETT plus BB group (*P* = 0.014, Table 2). There were no substantial differences in other secondary outcomes between the two groups, including the values of MAP and HR, the peak airway pressure and VT 5 min after OLV, the quality of lung isolation, and the duration from the end of surgery to the removal of laryngeal mask or endotracheal tube (Table 2). Few predefined potential intervention-related adverse events occurred overall, but only the incidence of hoarseness in the ETT plus BB group was significantly higher three days after operation compared with that in the SGA plus BB group (*P* = 0.036, Table 3).

**Table 3:** Adverse events during operation, during awakening and in 3 days after operation.

	SGA plus BB n = 60	ETT plus BB n = 60	P Value*
Adverse events			
During operation			
Hypoxemia	4/60 (6.7%)	6/60 (10.0%)	0.107
Hypercapnia	5/60 (8.3%)	3/60 (5.0%)	0.112
During awakening			
Laryngospasm	0/60	0/60	
Stridor	0/60	0/60	
Airway obstruction	0/60	0/60	
In 3 days after operation			
Hoarseness	0/60	5/60 (8.3%)	0.036

Values are expressed as number of patients/total patients (%).

Hypercapnia here specifically refers to the partial pressure of CO_2_ greater than 80 mmHg in such specific clinical context.

*P values indicate differences between SGA plus BB and ETT plus BB patients. *P* < 0.05 was considered statistically significant.

Abbreviations: SGA, supraglottic airway; ETT, endotracheal tube; BB bronchial blocker.

## 4 Discussion

Compared with the OLV method of ETT plus BB of extraluminal placement, the OLV of SGA plus BB method reduced the length of postoperative hospitalization stay and promoted the rapid recovery of patients. Additionally, this method was associated with adequate one lung ventilation, and fewer placement and positioning time. There were few if any significant adverse events in the intervention group (SGA plus BB) for OLV in children under the age of two years.

The complexity and uncertainty of thoracoscopic surgery for CPAM in children under the age of two years is ascribed to the internal space required for the operation created by shrinking the lung tissue under the condition of OLV or artificial pneumothorax. Creating an optimal environment for thoracoscopic surgery poses a great challenge for anesthesiologists and surgeons due to the bony structure of the thorax, the small thoracic space, intercostal stenosis, and bronchopulmonary variations. Fortunately, the pediatric surgery department at the Second Affiliated Hospital of Xi'an Jiaotong University is highly accomplished in pediatric lung surgery (about 300 thoracoscopic surgeries a year), and we have accumulated rich experience in anesthesia for pediatric thoracic surgery. OLV is a critical step in the successful completion of pediatric thoracic surgery because this procedure ensures an unobstructed airway, provides a clear surgical field of view, and protects the non-surgical lung from contamination by secretions and blood loss [[11], [12], [13]].

The methods of OLV include selective endobronchial intubation with a tracheal tube, double-lumen bronchial catheter ventilation, and BB [14]. Double-lumen bronchial catheter ventilation is only applicable to people over eight years old or weighing more than 30 kg because of the lack of suitable catheter sizes [15]. Although selective endobronchial intubation with a tracheal tube is suitable for OLV in children under the age of two years, this method cannot implement the free switching of OLV and double-lung ventilation during surgery which limits its application [16,17]. In the case of using BB, the small inner diameter of the endotracheal tube makes it difficult for the BB to be inserted through the lumen of endotracheal tube for children under two years old, thus limiting the intraluminal use of BB. The extraluminal placement of BB can be used for OLV in the children under two years old, but this may lead to the increased risk of laryngeal damage, longer procedural time, and accidental displacement [1,18]. Our colleagues at the Second Affiliated Hospital of Xi'an Jiaotong University have tried various methods of OLV for pediatric lung surgery carried out in the past few decades. After facingthe mentioned complications for a long time, an anesthesiologist accidently thought of a solution to conduct intraluminal use of BB through the SGA, and after urgent discussion with the medical committee, the procedure was put into practice immediately.

The combined use of SGA and BB for OLV has been used in adults, and was able to provide effective surgical exposure [8,19]. Furthermore, the combination also reduced the fluctuations of hemodynamic response, induced fewer airway injuries, and caused less post-operative sore throat symptoms and hoarseness [7,9]. However, the combination has not been used for OLV in lung surgery in children under the age of 2 years. The cuffless SGA used in this trial is designed to provide an efficient seal to the larynx without the inflatable cuff used in conventional SGAs for adults [20,21]. The positioning of SGA is easy, and the risk of tissue compression or dislodgement is low [22,23]. Thus, the SGA provides a useful alternative to endotracheal intubation, and is commonly used in pediatric anesthesia and served as a primary or back-up choice for difficult airway management [24]. Moreover, the length, opening angle, and inner diameter of SGA are benefit for bronchoscopy [25,26]. In this trial, we first placed the BB into the SGA, and then the BB was inserted into the glottis through the visual laryngoscope (Fig. 1). Eventually, the SGA was placed, and the fiberoptic bronchoscope was used by SGA to determine the position of the BB, which greatly shortened the placement and positioning time.

The SGA is easily placed, and does not directly stimulate the glottis and tracheal mucosa, which is beneficial to rapid recovery for patients [27,28]. In this trial, the length of postoperative hospitalization stay was less in the SGA plus BB group than that in the ETT plus BB group. Furthermore, the time for the placement and positioning of SGA plus intraluminal placement of BB was less than the time for placement and positioning of ETT plus extraluminal placement of BB. Meanwhile, the values of WBC and CRP on the first day of post-operation were less in the SGA plus BB group than that in the ETT plus BB group. This data supports that SGA plus BB may reduce the high-sensitivity stimulation after endotracheal intubation, decrease the complication rate of postoperative respiratory tract infection, and achieve rapid recovery after surgery. During the surgery, the peak airway pressure and VT values of the two groups were within the normal range, and there were no differences in the duration of OLV, the peak airway pressure, and VT values between the two groups, which indicated that the airway sealing of SGA was reliable and can meet changes of airway pressure during OLV. Meanwhile, few potentially predefined intervention-related adverse events occurred, but only the incidence of hoarseness of the ETT plus BB group was higher three days after operation compared with that in the SGA plus BB group.

When the method was first implemented, numerous questions arose, such as resolutions if the position of the SGA was not well placed, and what to do if the position of the laryngeal mask was displaced due to body position changes during or after surgery. We chose the extraluminal placement of BB with ETT as a back-up program if there were three failed attempts of SGA placement, which never happened during all surgeries. Once the SGA was placed, the anesthesiologists would secure it properly and ensure there were no shifts. When the patient was moved to a lateral decubitus position, the location of SGA and BB was checked again and adjusted if there were any positional changes. Moreover, an appropriate depth of anesthesia and muscle relaxation during surgery was maintained to keep the patient's position unchanged to prevent the displacement of SGA and BB. Before the end of surgery, the cuff of the BB was deflated and the collapsed lung segment was successfully re-expanded by several hand-controlled ventilations under thoracoscopic video.

We acknowledge that the most worrying aspect of laryngeal mask use is reflux aspiration, to which strict fasting and placement of a gastric tube can minimize its occurrence. As soon as regurgitation occurs, the patient's head should be immediately tilted to one side and the contents of the mouth should be immediately drawn away. Our trial met all the conditions for prevention of reflux aspiration because of routine preoperative fasting, use of laryngeal mask with esophageal drainage, and placement in a lateral decubitus position. Moreover, the anesthesiologists pay much attention to the corner of the mouth and intermittently carried out gastric aspiration during surgery. During the operation, the partial pressure of CO_2_ gradually increased, and the CO_2_ of nine patients exceeded 80 mmHg, one of whom was eliminated from this study because of intermittent suspension for bilateral lung ventilation to exhale CO_2_, resulting in surgery lasting more than 3 h.

In light of our promising results supporting a new procedural method for thoracoscopic surgery with OLV, there are still some limitations in our investigation. First, our method was performed as a single-center trial; thus, the findings might be influenced by the operators and the nursing team. Second, we did not collect much data on the parents of the patients’ nursing education and related psychological counseling, which might have an impact on our findings. Third, we did not design a full set of postoperative indicators of infection due to the reluctance to add additional financial and physical burden on the patients. While we obtained the values of WBC and CRP on the first day of post-operation following our medical routine of surgical postoperative management, collection of more parameters could further support our study. If a computed tomography (CT) examination or the better choice of a lung ultrasound, and indicators of infection at more time points were conducted, the correlation between the intraluminal placement of the BB with SGA and the shortened postoperative hospital stay could add more clarity to our results. In our ongoing follow-up study, we are addressing these issues.

## 5 Conclusion

There were few if any significant adverse events in the intervention group (SGA plus BB) for OLV in children under the age of two years, which resulted in a shorter postoperative hospitalization stay compared to the control group (ETT plus BB). Therefore, the SGA plus BB method seems to be worthy of wider clinical application for other patients with different characteristics and patients in other hospitals. Further investigation of the mechanism for this novel technique to shorten the length of postoperative hospitalization stay would promote our novel technique.

## Author contribution statement

Junlin Lv: Performed the experiments.

Xiaoying Ding; Jing Zhao: Analyzed and interpreted the data.

Huijuan Zhang; Jiaojiao He: Contributed reagents, materials, analysis tools or data.

Lei Ma: Conceived and designed the experiments; Wrote the paper.

Jianrui Lv: Conceived and designed the experiments.

## Funding statement

Lei Ma was supported by 10.13039/501100001809National Natural Science Foundation of China [81601148], 10.13039/501100007128Natural Science Foundation of Shaanxi Province [2022JM-488].

## Data availability statement

Data will be made available on request.

## Declaration of interest's statement

The authors declare no competing interests.
